# Decreased CD90 expression in human mesenchymal stem cells by applying mechanical stimulation

**DOI:** 10.1186/1746-160X-2-8

**Published:** 2006-03-31

**Authors:** Anne Wiesmann, Hans-Jörg Bühring, Christoph Mentrup, Hans-Peter Wiesmann

**Affiliations:** 1 Medizinische Klinik und Poliklinik, Abteilung für Hämatologie, Onkologie, Immunologie und Rheumatologie, Universitätsklinikum Tübingen, Otfried-Müller-Str. 10, D-72076 Tübingen, Germany; 2 Klinik und Poliklinik für Mund und Kiefer-Gesichtschirurgie, Westfälische Wilhelms-Universität Münster, D-48149 Münster, Germany

## Abstract

**Background:**

Mesenchymal stem cells (MSC) are multipotent cells which can differentiate along osteogenic, chondrogenic, and adipogenic lineages. The present study was designed to investigate the influence of mechanical force as a specific physiological stress on the differentiation of (MSC) to osteoblast-like cells.

**Methods:**

Human MSC were cultured in osteoinductive medium with or without cyclic uniaxial mechanical stimulation (2000 μstrain, 200 cycles per day, 1 Hz). Cultured cells were analysed for expression of collagen type I, osteocalcin, osteonectin, and CD90. To evaluate the biomineral formation the content of bound calcium in the cultures was determined.

**Results:**

After 14 days in culture immunfluorescence staining revealed enhancement of collagen type I and osteonectin expression in response to mechanical stimulation. In contrast, mechanically stimulated cultures stained negative for CD90. In stimulated and unstimulated cultures an increase in the calcium content over time was observed. After 21 days in culture the calcium content in mechanical stimulated cultures was significantly higher compared to unstimulated control cultures.

**Conclusion:**

These results demonstrate the influence of mechanical force on the differentiation of human MSC into osteoblast-like cells in vitro. While significant enhancement of the biomineral formation by mechanical stimulation is not detected before 21 days, effects on the extracellular matrix became already obvious after 14 days. The decrease of CD90 expression in mechanically stimulated cultures compared to unstimulated control cultures suggests that CD90 is only transiently expressed expression during the differentiation of MSC to osteoblast-like cells in culture.

## Background

Mesenchymal stem cells (MSC) are pluripotent cells with the ability to differentiate along osteogenic, chondrogenic, and adipogenic lineages [1]. MSC, first described by Friedenstein [2], have also been denoted as mesenchymal progenitor cells, fibroblast colony-forming units, colony-forming unit-fibroblasts and marrow stromal cells. The best studied and accessible source of MSC is the adult bone marrow. In contrast to hematopoietic stem cells (HSC), MSC lack an unique surface antigen for positive selection. Identification of MSC is based on differentiation properties and an extensive panel of monoclonal antibodies, including differentiation and lineage specific markers, growth factor receptors, and adhesion molecules.

MSC are suggested to be positive for CD73 (SH3 and SH4), CD105 (SH2), CD29, CD44, CD90, and CD166 and negative for CD14, CD34, CD38, CD45. In addition, there is evidence that MSC change their expression pattern of surface marker proteins depending on culture time. To add further confusion to the characteristics of MSC, it appears that the source of harvested MSC plays a role in lineage commitment [3]. Conflicting results in the literature may be due to different isolation protocols resulting in heterogeneous cell populations of immature stem cells and more restricted progenitor cells.

Differentiation of MSC in vitro can be assessed by lineage specific protein expression.

The ability to direct MSC towards an osteogenic phenotype plays a key role in regenerative medicine[4]. Furthermore, recent data underline the importance of osteoblasts for the hematopoietic stem cell niche [5,6]. Osteogenic differentiation of MSC, which can be induced by the addition of dexamethasone, β-glycerophosphate and ascorbate [7], is generally assed by monitoring collagen type I, osteocalcin and alkaline phosphatase (ALP) expression. Furthermore, CD90 (Thy-1) was discussed to be useful as a differentiation marker in following the development of osteoblasts. The expression of this 25–30 kDa GPI-linked membrane protein, which precise biological function is not clear yet, was described to decline as the osteoblast matures [8].

In addition, biomineral formation in MSC cultures serves as an indicator for differentiation into osteoblast-like cells and physiological mechanical stimulation has been described to enhance mineralization and to induce osteogenic differentiation of MSC in vitro and in vivo [9,10].

To investigate the influence of physiological uniaxial mechanical stimulation on the differentiation of human MSC into osteoblast-like cells, calcium concentration and the expression of marker proteins such as collagen type I and II, osteocalcin and CD90 were determined in mechanically stimulated cultures and unstimulated control cultures.

## Materials and methods

### Mesenchymal stem cells

Human mesenchymal stem cells (hMSC) were purchased from Cambrex (Cambrex Bio Science, Verviers, Belgium). For seeding, subculturing and differentiation the instructions and the chemicals of the manufacturer were followed.

Briefly, the cryopreserved cells were quickly thawed at 37°C, diluted with mesenchymal stem cell growth medium, centrifuged, resuspended in medium and seeded at a density of 5000 cells/cm^2^. Three days after plating the cells were fed. After 6 or 7 days the cells were nearly confluent and were detached with trypsin-EDTA.

For mechanical stimulation the cells were seeded onto polycarbonate carriers at a density of 50000 cells/cm^2^. The cells were maintained in complete osteogenesis induction medium containing dexamethasone, ascorbic acid and β-glycerophosphate. The media was changed every three days.

### Application of mechanical stress

The application of mechanical stress on the cells was conducted with a specially designed 4-point bending device [16]. With this instrument the cell layer on the polycarbonate carrier was subjected to homogeneously distributed uniaxial bending stimuli. The applied uniaxial bending stimuli were measured in strain. Strain is the quotient of the difference of the length of the surface with and without application of bending stimuli divided by the length without the stimulus (1.000 μstrain equal an elongation of 0,1%, 10.000 μstrain equal an elongation of 1%). The applied values were 0 and 2.000 μstrain. For the duration of the experiment the twist was applied at 200 cycles per day at a frequency of 1 Hz. The application of mechanical stress started 3 days after the cells were seeded onto the carriers. Control cultures were maintained under identical culture conditions without mechanical stimulation.

### Calcium determination

The bound calcium was determined spectroscopically. After decanting the medium the cell-layer was washed twice with PBS (pH 7.4). To dissolve the mineral 4 ml 0.1 M HCl were added. The calcium concentration was measured spectroscopically at 600 nm by using a calorimetric assay (Arsenazo III, Sigma-Aldrich, München, Germany) and a standard solution containing calcium (10 mg/dl Calcium, München, Sigma-Aldrich, Germany) for calibration.

### Immunfluorescence

The cell layer was washed twice with PBS (pH 7.4), fixed for 10 min with 100% methanol at -20°C and left to air dry. For assessment of the expression of proteins the following antibodies were applied: monoclonal anti-osteocalcin and anti-osteonectin (Takara Biochemical Europe S.A., Gennevielliers, France) and polyclonal anti-collagen type I (BioTrend Chemikalien GmbH, Köln, Germany), and monoclonal anti-CD 90 (BD Biosciences, Heidelberg, Germany). Fluorochrome-conjugated secondary antibodies (Mobitec GmbH, Göttingen, Germany) were used for staining.

### Immunhistochemistry

The cell layers were processed as above (Immunfluorescence) with the exception that a peroxidase labeled DAKO Envision detection system (DAKO, Diagnostics AG, Hamburg, Germany) with AEC as substrate was used instead of the fluorochrome-conjugated secondary antibodies. For assessment of the expression of proteins the following antibodies were applied: polyclonal anti-collagen II (Quartett Immundiagnostika und Biotechnologie GmbH, Berlin, Germany), polyclonal anti-collagen I (BioTrend Chemikalien GmbH, Köln, Germany) and monoclonal anti-osteocalcin (Takara Biochemical Europe S.A., Gennevielliers, France). For an overview the cells were stained for 2 min at 60°C with an aqueous solution of Toluidin Blue O 2,5% (w/v).

### Statistical analysis

Means and standard deviations (S.D.) were calculated for descriptive statistical documentation. The unpaired Student's t-test was applied for analytical statistics. A value of p < 0.05 was considered significant.

## Results

Mesenchymal stem cells were cultivated for 21 days in osteoinduction medium. Cultured cells were mechanically stimulated 200 times per day with a cyclic uniaxial mechanical stimulus of 2000 μstrain. A control group was maintained under the same culture conditions without mechanical stimulation.

**Figure 1:**
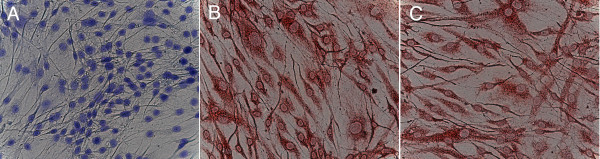
**Immunhistochemical staining of cultured human MSC**. MSC were cultured for 14 days in osteoinduction medium. Uniaxial bending stimuli were applied as described. Cultures were stained with A: Toluidin Blue; B: anti-collagen type I; C: anti-osteocalcin.

After 14 days in medium, mechanically stimulated cultures stained positive for osteoblastic specific markers like collagen type I and osteocalcin even without showing the typical osteoblast morphology (Figure 1).

**Figure 2:**
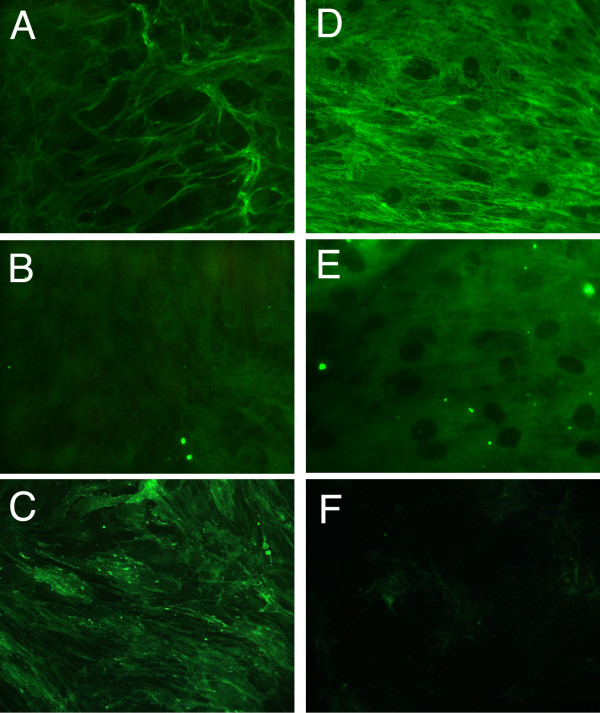
**Immunfluorescence staining of cultured human MSC. after 14 days of osteogenic stimulation**. A-C: MSC were cultured for 14 days in osteoinduction medium containing dexamethasone, ascorbate and β-glycerophosphate. D – F: Cultures stimulated with osteoinduction medium and mechanical stimulation. Cultures stained with: A and D: anti-collagen I; B and E: anti-osteonectin; C and F: anti-CD90.

Immunfluorescence staining of cultured MSC is summarized in Figure 2. Cultured cells without mechanical stimulation stained positive for collagen type I, but additional mechanical stress resulted in an enhanced expression (Figure 2D). Compared to collagen type I expression, staining for osteonectin was weak for cultured cells without mechanical stimulation and could be enhanced marginally by bending stimuli (Figure 2B and 2E). In contrast, cultured cells without mechanical stimulation stained positive for CD90, whereas cultured cells showed no CD90 expression when mechanical stress was applied. Therefore, mechanical stress resulted in a marked decrease of CD90 expression.

**Figure 3:**
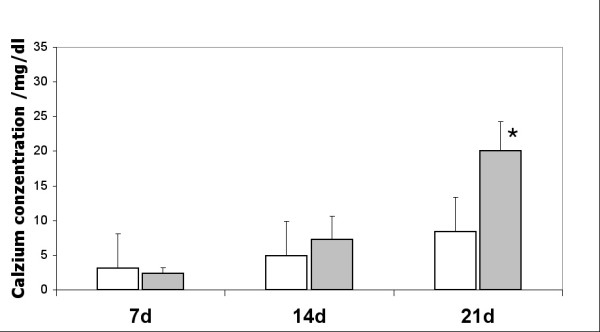
**Concentration of bound calcium in human MCS cultures**. Grey bars correspond to cultures cultivated in osteoinduction medium containing dexamethasone, ascorbate and β-glycerophosphate with uniaxial mechanical stimulation (2000 μstrain, 200 × day, 1 Hz). White bars correspond to control cultures without mechanical stimulation. The calcium concentration was measured at day 7, 14 and 21. Statistically significant differences between mechanically stimulated and mechanically unstimulated cultures are indicated by *.

The calcium concentrations in the mechanically stimulated and unstimulated cultures were determined after 7, 14 and 21 days in culture. The results are presented in Fig. 3. For stimulated and unstimulated cultures an increase in the calcium concentration could be observed. This increase is more prominent for the mechanically stimulated cultures. After 7 days of culture there is no difference in calcium concentration between mechanically unstimulated and mechanically stimulated cultures. In contrast, after 3 weeks in culture, there is a significant higher calcium concentration in mechanically stimulated cultures compared to mechanically unstimulated cultures.

## Discussion

MSC are known to be capable to differentiate into osteoblast-like cells, which respond to physiological mechanical loads in vivo and in vitro [11-13]. The most widely used mechanical stimuli in vitro are cyclic stretch and fluid shear flow [14]. Since the experiments differ in the applied mechanical stimuli (e. g. cyclic uniaxial, cyclic biaxial, fluid flow), the duration, applied forces and frequency of stimulation and in the isolated cell populations comparison of the results is difficult. Studies directly comparing the influence of these parameters are rare [15,16].

The production of mineralised matrix is considered as a marker for terminally differentiated MSC into osteoblast-like cells [10,17]. Therefore, mineral formation is an appropriate indicator whether mechanical stimulation accelerates osteogenic differentiation of MSC. In this work uniaxial mechanical load (2000 μstrain, 200 × per day, 1 Hz) enhanced the biomineral formation over time. Mineral formation in mechanically stimulated cultures was significantly enhanced after 21 days compared to unstimulated control cultures. This result is in accordance to other reports, which demonstrated a significant increase of mineral formation by mechanical stimulation. Simmons et al. showed an enhanced mineral formation in stimulated cells compared to unstimulated cells after 9 days [9]. The cells were strained continuously at 0.25 Hz with an equibiaxial cyclic strain of 3%. In another experiment rat marrow stromal cells were seeded in a 3D culture and subjected to fluid flow of 0,3 ml/min or more [18]. After 16 days mechanical stimulation significantly increased the calcium content of the culture. Therefore, differentiation of MSC towards osteoblast-like cells can be influenced by different mechanical loading.

Prior to mineralization the effects of the mechanical stimulation become obvious by analysis of the extracellular matrix. As the most abundant extracellular protein and location of mineralization the collagen type I expression was analysed by immunfluorescence. The expression of this protein is upregulated in the early phase of osteoblastic differentiation and Takano et al. have shown, that mechanical strain affects the collagen type I microarchitecture in bone tissue [19]. In this study collagen type I expression was enhanced by mechanical stimulation. Another group demonstrated, that collagen type I itself induces the differentiation of osteoprogenitor cells into osteoblast-like cells [20]. Therefore, increased collagen type I expression in the mechanically stimulated cultures may function as a positive feedback for differentiating MSC in culture.

The maturation of the collagen matrix was evaluated by the osteonectin and osteocalcin expression. The expression of osteonectin is limited to cells associated immediately with mineralized tissues and it is thought to mediate deposition of hydroxyapatite [21]. The expression of osteonectin was enhanced by mechanical stimulation after 14 days. A similar result was reported earlier for a culture of primary osteoblast-like cells under similar experimental conditions (Meyer 01). Therefore the enhanced osteopontin expression in this work not only documents the accelerated maturation and therefore differentiation of the mechanically stimulated MSC but also indicates the cultured cells as osteoblast-like cells.

In contrast to osteonectin, osteocalcin, which is expressed shortly before mineralization, is a late marker for differentiation. Its presence has been considered to establish the differentiated state of the osteoblast [22,23]. Interestingly, even MSC cultures with a fibroblast-like stretched spindle shaped morphology, which is atypical for osteoblast-like cells, were demonstrated to express osteocalcin after 14 days in culture with mechanical stimulation.

Another antigen evaluated in this study is CD90 (Thy-1), which is commonly used as a positive marker for MSC. In addition, CD90 has been described as a possible marker for osteoblastic differentiation [8]. In this study CD 90 was expressed on MSC cultured for 14 days in osteoinductive medium. When cultured cells were stimulated mechanically, CD90 expression decreased while there was an increase of collagen I and osteonectin protein expression. Therefore, it is likely that CD90 is expressed during proliferation but expression level declines as the cells mature towards osteoblast-like cells. CD90 could then be considered as a transient marker for early MSC differentiation towards osteogenic cells. Additional experiments (expression studies of CD90 during in vitro culture of mechanically stimulated MSC) will further evaluate the role of CD90 during differentiation of MSC.

## Conclusion

This study demonstrated that uniaxial mechanical loads (2000 μstrain, 200 × per day, 1 Hz) of in vitro cultured MSC enhanced the collagen type I and osteonectin expression after 14 days compared to an unstimulated control. Moreover the expression of CD90 (Thy-1) was decreased by mechanical stimulation after 14 days. The CD90 expression during MSC differentiation might therefore be useful as a transient marker for MSC differentiation.

After 21 days of mechanical stimulation, an increase in matrix bound mineral formation was detected indicating that uniaxial mechanical stimulation is an appropriate stimulator for differentiation of MSC into osteoblast-like cells. The mineral formation together with an osteocalcin expression indicates the osteoblast-like nature of the differentiated MSC.

## Competing interests

The author(s) declare that they have no competing interests.
